# The Novel Angiotensin-(1–7) Analog, A-1317, Improves Insulin Resistance by Restoring Pancreatic *β*-Cell Functionality in Rats With Metabolic Syndrome

**DOI:** 10.3389/fphar.2020.01263

**Published:** 2020-08-24

**Authors:** Maria Andréa Barbosa, Claudiane Maria Barbosa, Taynara Carolina Lima, Robson Augusto Souza dos Santos, Andréia Carvalho Alzamora

**Affiliations:** ^1^Núcleo de Pesquisa em Ciências Biológicas, Universidade Federal de Ouro Preto, Ouro Preto, Brazil; ^2^Departamento de Fisiologia e Biofísica, Universidade Federal de Minas Gerais, Belo Horizonte, Brazil; ^3^Departamento de Ciências Biológicas, Instituto de Ciências Exatas e Biológicas, Ouro Preto, Brazil

**Keywords:** metabolic syndrome, angiotensin-(1–7), A-1317, liver metabolism, Mas, Mas-related G protein-coupled receptor member D, adenosine monophosphate activated protein kinase, pancreatic *β*-cell

## Abstract

In previous studies we have shown that oral Ang-(1–7) has a beneficial therapeutic effect on cardiometabolic disturbances present in metabolic syndrome (MetS). Based on the fact that Ang-(1–7) acts through release of nitric oxide (NO), a new peptide, A-1317 was engineered adding the amino acid L-Arginine, the NO precursor, to the N-terminal portion of the Ang-(1–7). Therefore, in a single molecule the substrate and the activator of NO are combined. In the present study, we evaluated the effect of A-1317 oral treatment on liver-glucose metabolism in MetS induced by high fat (HF) diet in rats. Rats were subjected to control (AIN-93M, CT) or HF diets for 15 weeks to induce MetS and treated with A-1317, Ang-(1–7) included into hydroxypropyl-*β*-cyclodextrin (HP*β*CD) or empty HP*β*CD (E), in the last 7 weeks. At the end of 15 weeks, hemodynamic, biometric, and biochemical parameters, redox process, and qRT-PCR gene expression of NO synthase and RAS components were evaluated in the liver. HF/E rats increased body mass gain, adiposity index, despite the reduction in food intake, increased plasma leptin, total cholesterol, triglycerides, ALT, fasting blood glucose, OGTT and insulin, HOMA-IR and MAP and HR. Furthermore, the MetS rats presented increased in liver *angiotensinogen*, *AT1R*, *ACE* mRNA gene expression and concentration of MDA and carbonylated protein. Both Ang-(1–7) and A-1317 oral treatment in MetS rats reverted most of these alterations. However, A-1317 was more efficient in reducing body mass gain, ALT, AST, total cholesterol, insulin, fasting blood glucose, ameliorating *β* cell capacity by increasing HOMA-*β* and QUICKI, whereas Ang-(1–7) reduced HOMA-*β* and QUICKI. In addition, Ang-(1–7) increased *Mas* and *AKT* liver mRNA gene expression, while A-1317 increased both *Mas* and *MRGD* and *AMPK* liver mRNA gene expression, suggesting a distinct pathway of action of Ang-(1–7) and A-1317 in MetS rats. Taken together, our data showed that treatment with A-1317 was able to ameliorate MetS disorders and suggested that this effect was mainly *via* MRGD *via* activation of AMPK and increasing *β* cell function.

## Introduction

High fat diet (HF) and/or physical inactivity can induce metabolic syndrome (MetS) which is characterized by cardiometabolic disorders such as insulin resistance (IR), high blood pressure and central obesity (Alberti et al., 2009; Zacarias et al., 2017; Barbosa et al., 2019). These disorders are related to redox imbalance resulting in higher formation of reactive oxygen species such as superoxide and hydroxyl radicals, which can lead to mitochondrial dysfunction and accumulation of oxidized proteins and lipids (Estadella et al., 2013; Peluso et al., 2017; Zacarias et al., 2017; Vona et al., 2019). In addition, HF diet induces liver damage related to various disorders occurring in the MetS such as IR, dyslipidemia, and increased adiposity (Rosselli et al., 2014; Zacarias et al., 2017; Barbosa et al., 2019; Figueiredo et al., 2019). Additionally, in pancreatic *b*-cells, different concentrations of nitric oxide (NO) exerts positive or negative regulation of insulin secretion (Meares et al., 2011) and thus improving or worsening the state of IR (Kaneko and Ishikawa, 2013). Furthermore, some studies have suggested that adenosine monophosphate activated protein kinase (AMPK), an enzyme that plays a role in cellular energy homeostasis, acts as a negative regulator of insulin secretion in pancreatic *β*-cells (Fu et al., 2013), regulating NO bioavailability by phosphorylating eNOS (Meares et al., 2011; Patel et al., 2016).

In the renin angiotensin system (RAS), the angiotensin-converting enzyme 2 (ACE2)/angiotensin-(1–7) [Ang-(1–7)]/Mas-related G protein-coupled receptor (Mas) axis is a counter-regulator of the actions of the angiotensin-converting enzyme (ACE)/angiotensin II (Ang II)/angiotensin II type 1 receptor (AT1R) axis in different disease states (Dominici et al., 2014; Santos et al., 2018). Ang-(1–7) through activation of Mas (Santos et al., 2003) acts mainly through NO and protein kinase B (AKT), a serine/threonine specific protein kinase, that plays a key role in glucose metabolism, apoptosis, cell proliferation, transcription, and cell migration being suggested as a therapeutic strategy for cardiometabolic diseases (Sampaio et al., 2007; Dias-Peixoto et al., 2008; Whitaker and Molina, 2014; Santos et al., 2018). On the other hand, studies show that in addition to Mas, some effects of Ang-(1–7) can also be mediated through Mas-related G protein-coupled receptor member D (MRGD) (Tetzner et al., 2016; Gunarathne et al., 2019) *via* AMPK (Liu et al., 2019).

In prevention studies, oral administration of Ang-(1–7) improved body mass, adiposity index, plasma triglycerides levels, glucose tolerance, insulin sensitivity in rats and mice fed with HF (Oliveira Andrade et al., 2014; Williams et al., 2016) and fructose (Giani et al., 2009; Muñoz et al., 2012) diets. Additionally, in recent studies (Barbosa et al., 2019; Figueiredo et al., 2019) we have shown that oral treatment with Ang-(1–7) in rats, with established MetS induced by HF diet, was effective in restoring biometric, biochemical parameters, redox process, and RAS components in the liver and gastrocnemic muscle (Figueiredo et al., 2019). Also, oral treatment with Ang-(1–7) was able to remodel the white and brown adipose tissue (Barbosa et al., 2019). This data set shows the effectiveness of oral Ang-(1–7) treatment in restoring different MetS disorders already established in rats.

Given the evidence that Ang-(1–7) treatment is efficient in the treatment of MetS and that these benefits may occur through NO, here we studied whether the addition of L-Arginine in the N-terminal portion of Ang-(1–7), A-1317 compound, could enhance the beneficial effect observed by Ang-(1–7) in the already established MetS. This compound was previously used in our laboratory for treatment of muscular dystrophy (Oliveira, 2019).

## Methods

### Animals

The study used male Fischer rats, aged 4 weeks (71.7 ± 1.04 g, n = 60), from the Animal Science Center (CCA/UFOP) of the Federal University of Ouro Preto (UFOP, Brazil). The animals were kept in individual cages under controlled temperature (25 ± 1°C) and a light–dark cycle of 12 h–12 h. Throughout the experiment, the animals had free access to water and diet. All procedures were performed in accordance with the Guidelines for Ethics in Care of Experimental Animals. The project was approved by the animal ethics committee of the Federal University of Ouro Preto protocol 2016/51.

### Study Design

After weaning, the animals were fed with control diet (AIN-93 M) or high fat diet (HF, 37% lard, **Table 1**) for 15 weeks and food intake was evaluated weekly. In the eight week of diets, MetS effects were analyzed on body mass, fasting glucose, plasma levels of total cholesterol, high density lipoprotein (HDL), and triacylglycerol using commercial kits (Labtest, Lagoa Santa, MG, Brazil). The mean arterial pressure (MAP) and heart rate (HR) were evaluated by digital tail plethysmography (Panlab, LE5001). Additionally, oral glucose tolerance test (OGTT) was performed by assessing glycemia by gavage administration of 40% glucose solution (1 g/kg) to the animals after 10, 20, 30, 60, 90, and 120 min by glucose analysis (glycosimeter, Accu-chek^®^), and the area under the curve (AUC) was calculated using trapezoidal analysis (Song et al., 2004). After eight weeks of the diets, orally by gavage, the treatment with HP*β*CD/A-1317 (42 μg/kg/day), HP*β*CD/Ang-(1–7) (40 μg/kg/day) or HP*β*CD/Empty [HP*β*CD without the inclusion of Ang-(1–7) or A-1317] was started for seven weeks. At the end of the 15 weeks, blood samples (fasting 12 h) were collected and centrifuged (8.000g, 4°C, 6 min), and serum was aliquoted and stored at (−80°C) for the biochemical analyses. Liver and retroperitoneal, epididimal and inguinal fat deposits were removed, weighed (g/100 g rat mass), placed in liquid nitrogen and stored at −80°C for qRT-PCR, oxidative stress and RAS component evaluations. Adiposity index was measured by the formula [inguinal fat deposit + epididymal fat deposit + retroperitoneal fat deposit absolute × 100 (Barbosa et al., 2018). The experimental groups were: 1) CT/E (n = 10): rats fed with CT diet and treated with empty HP*β*CD during the last seven weeks of diet. 2) CT/Ang-(1–7) (n = 10): rats fed with CT diet and treated with HP*β*CD/Ang-(1–7) during the last seven weeks of diet; 3) CT/A-1317 (n = 10): rats fed with CT diet and treated with HP*β*CD/A-1317 during the last seven weeks of diet; 4) HF/E (n = 10): rats fed with HF diet and treated with empty HP*β*CD during the last seven weeks of diet; 5) HF/Ang-(1–7) (n = 10): rats fed with HF diet and treated with HP*β*CD/Ang-(1–7) during the last seven weeks of diet; 6) HF/A-1317 (n = 10): rats fed with HF diet and treated with HP*β*CD/A-1317 during the last seven weeks of diet.

**Table 1:** Composition and energy content of diets.

Ingredients (g/kg)	Control AIN-93M	High-fat diet
Corn starch	620.70	–
Sucrose	100.00	–
Fructose	–	34.2
Casein	140.00	180.50
Condensed milk	–	316.00
Soybean oil	40.00	–
Lard	–	370.00
Fiber (cellulose)	50.00	50.00
Wheat bran	–	–
Mineral mix (AIN-93G-MX)*	–	35.00
Mineral mix (AIN-93M-MX)*	35.00	–
Vitamin mix (AIN-93G-VX)*	10.00	10.00
DL-Methionine	1.80	3.00
Choline Chloride	2.50	2.50
**Macronutrients (% by weight)**		
Protein	14.00	20.26
Carbohydrate	72.07	20.68
Fat	4.00	39.53
**Macronutrients (% kcal)**		
Protein	14.72	15.60
Carbohydrate	75.82	15.92
Fat	9.46	68.48
Saturated	15.20	28.09
Monounsaturated	23.30	28.46
Polyunsaturated	60.00	8.87
**kcal/g**	3.80	5.20
**kj/g**	15.91	21.77

Composition of diets (g/kg) consumed by rats. The percentage of kcal was calculated based on the calories provided by each macronutrient, as follows: carbohydrates 4 kcal/g; 4 kcal proteins/g; and lipids 9 kcal/g. The percentage of polyunsaturated, monounsaturated, and saturated fats was calculated according to the total amount of lipids (g) supplied in each diet. (*) mixture of vitamins.

### Biochemical Analysis

At the end of 15 weeks, blood samples (fasting 12 h) were collected and centrifuged (8.000g, 4°C, 6 min) to separate the serum for determination of fasting glucose, total cholesterol, low-density lipoprotein (LDL), high-density lipoprotein (HDL), triacylglycerol, albumin, total proteins, alanine aminotransferase (ALT), and aspartate aminotransferase (AST). The analyses were performed using individual commercial kits (Labtest, Lagoa Santa, MG, Brasil) according to the instructions provided by the manufacturer. Analyses were performed in the Pilot Laboratory of Clinical Analyses (LAPAC/UFOP). Model assessment of IR homeostasis was calculated (HOMA-IR) = fasting insulin (μIU/ml) × fasting glucose (mmol/ml)/22.5 and a model for assessing homeostasis of the functional capacity of the *β* cells (HOMA-*β*) = 20 × fasting insulin (μIU/ml)/fasting glucose (mmol/ml) − 3.5 (Matthews et al., 1985), quantitative insulin sensitivity check index (QUICKI) = 1/[LOG [fasting insulin (μIU/ml)]/LOG [fasting glucose (mmol/ml)] (Sarafidis et al., 2007). Insulin and leptin levels were determined by the Elisa sandwich type immunoassay method using the Ultra-sensitive Rat Elisa Kit (Crystal Chem, Downers Grove, IL., USA) according to the instructions provided by the manufacturer.

### Superoxide Dismutase Activity

Frozen liver samples (100 mg) were homogenized in phosphate buffer (pH 7.4) and centrifuged at 12,000g for 10 min at 4°C. The activity of the enzyme superoxide dismutase (SOD) was evaluated indirectly with an ELISA reader at 570 nm, based on the ability of this enzyme to eliminate the superoxide anion, decreasing the reduction of thiazolyl blue tetrazolium bromide and converting superoxide anion to hydrogen peroxide and thereby reducing the auto oxidation rate of pyrogallol (Dieterich et al., 2000). The results were expressed as U/mg protein, in which a unit of SOD is defined as the amount of enzyme required for 50% inhibition of thiazolyl blue tetrazolium bromide reduction.

### Thiobarbituric Acid-Reactive Substances and Carbonyl Protein

Malondialdehyde (MDA) concentrations were determined using thiobarbituric acid reactive substance (TBARS) concentrations. Liver samples (100 mg) were homogenized in KPE (potassium phosphate–EDTA) buffer (pH 7.4) and centrifuged (10,000g, 10 min, at 4°C). The supernatant was collected and used as the biological sample. Briefly, the samples from the homogenates were mixed with 1 ml of 10% trichloroacetic acid and 1 ml of 0.67% thiobarbituric acid and then heated in a boiling water bath for 30 min. TBARS was determined from the absorbance at 532 nm. The absorbance of the supernatant was determined at 370 nm. Both series of data are expressed in nmol/mg of protein. The contents of carbonylated protein were determined according to the method of Levine et al. (1990). Data were expressed as nmol/mg protein.

### Gene Expression Analysis

In separated groups of rats, qRT-PCR was performed in the liver. The total RNA from the liver was isolated with TRI reagent^®^ (Sigma-Aldrich) according to the manufacturer’s protocol. All isolated RNA was quantified by spectrophotometry, and the optical density was estimated from the 260/280 nm absorbance ratio. A reverse transcriptase reaction was performed using SuperScript ™ III (Invitrogen Life Technologies) for first-strand cDNA synthesis. Real-time PCR was carried out following the generation of first-strand cDNA. A PCR for each sample was carried out in triplicate for all cDNAs and for the 18s ribosomal control and were used SYBR^®^ Green PCR Master Mix (Applied Biosystems, Rockford, USA). The analyzed genes are described in **Table 2**. The analyses were performed by a relative method for quantifying gene expression (comparative Cq, ΔCq), which allows one to quantify differences among samples in the level of expression of a specific target. The expression levels were normalized for the amount of the reference gene (*Rplp2*) on each plate. The results were obtained with a formula that considers the amount of the target gene normalized to the calibrator gene, given by (2–ΔCq).

**Table 2:** Rat Genome Database (RGD) accession numbers and primer sequences of genes selected for qRT-PCR.

Gene	Accession number (RGDID)	Primer sequence (5′–3′)
***Angiotensinogen***	134432.2	F 5′-CTGTGAAGGAGGGAGACTGC-3′ R5′-CAG CAA GCC CTG ACC AGC-3′
***ACE***	012544.1	F 5′-TGGCACTTGTCTGTCACTGG-3′ R 5′-ACACCCAAAGCA ATTCTTCG-3′
***Agtr1a/b (AT1R)***	030985.4	F 5′-TCACTTTCCTGGATGTGCTG-3′ R 5′-GATGGGCATGGCAGTGTC-3′
**A*CE2***	001012006.1	F 5′-GAGATGAAGCGGGAGATCG-3′ R 5′-TGGAACAGAGATGCAGGGTC-3′
***Mas***	012757.2	F 5′-CAATCGTGACGTTATCGGTG-3′ R 5′-TCTCTCCACACTGATGGCTG-3′
***AKT***	016988.2	F 5′-GGAGGTCATGGAGCATCGGTTC-3′ R 5′-GTTTGAAGGGTGGCAGGAGC-3′
***AMPK***	023991.1	F 5′-GAAGATCGGACACTACGTGCT-3′ R 5′-CTGCCACTTTATGGCCTGTC-3′
***MRGD***	001001506.1	F 5′- TGTGTGGGATAGTGGGCAAC-3′ R 5′- TGAGCACGTAGGTGCAGAAG-3′
***eNOS***	021838.2	F 5′-CATACTTGAGGATGTGGCTGTC -3′ R 5′-CCACTGCTGCCTTGTCTTTC -3′
***iNOS***	012611.3	F 5′-CTGGGCTGTGCAAACCTTC -3′ R 5′-AGCGTTTCGGGATCTGAATG -3′
***Rplp2***	001108150	F 5′-TACTAGACAGCGTGGGCATC-3′ R 5′-CAACACCCTGAGCGATGACA-3′

Primers used (Forward and Reverse).

### Statistical Analysis

Results are expressed as means ± SEM. Data were analyzed for Kolmogorov–Smirnov normality and followed the standard normal distribution. After, they were evaluated by two-way ANOVA, followed by Tukey’s post-test. Statistical analyzes were performed with GraphPad Prism software (version 6.0, San Diego, USA). Statistical significance was set at p <0.05.

## Results

### Evaluation of MetS Establishment

After eight weeks of diets, HF rats showed characteristic disorders of the MetS such as increased body mass, fasting blood glucose levels, glucose area under the curve (AUC), serum triacylglycerol, MAP, HR, and reduction in HDL cholesterol levels compared to CT rats. There was no significant difference in total cholesterol levels in all the evaluated groups (**Table 3**).

**Table 3:** Evaluation of MetS establishment in rats after 8 weeks of HF diet.

Parameters	CT/E	HF/E
Body mass (g)	264 ± 3.1	307 ± 6.8*
Blood glucose level (mg/dl)	124.9 ± 5.344	172.7 ± 9.242*
OGTT (AUC)	14460 ± 415.6	16594 ± 441.2*
Total cholesterol (mg/dl)	57.8 ± 3.4	64.0 ± 4.6
HDL cholesterol (mg/dl)	37.6 ± 1.7	31.27 ± 0.9*
Triacylglycerol (mg/dl)	41.4 ± 3.7	59.2 ± 3.9*
MAP (mmHg)	108.4 ± 2.1	121.4 ± 5.1*
HR (beats/min)	384.4 ± 11.3	418.0 ± 3.9*
n	9-20	9-20

*p < 0.05 compared to CT/E group. Values are expressed as mean values ± standard error of the mean and analyzed using unpaired student t-test. OGTT, oral glucose tolerance test; AUC, area under the curve; MAP, mean arterial pressure; HR, heart rate; n = number of animals.

### Oral A-1317 Treatment Decreases Body Mass Gain

HF/E, HF/Ang-(1–7) and HF/A-1317 rats showed decreased in food intake and no difference in caloric intake compared to rats CT/E. However, oral treatments with Ang-(1–7) or A-1317 decreased the adiposity index compared to HF/E rats. In addition, only treatment with A-1317 decreased body mass gain compared to HF/E rats. There was no difference in liver mass in all groups (**Table 4**).

**Table 4:** Feed intake and biometric parameters of rats fed with CT or HF for 15 weeks and treated with HP*β*CD/A-1317 or HP*β*CD/Ang-(1-7) during the last 7 weeks of the diet.

Parameters	Experimental Groups					
	CT/E	CT/Ang-(1–7)	CT/A-1317	HF/E	HF/Ang-(1–7)	HF/A-1317
Food intake (g)	92.0 ± 4.3	82.4 ± 1.4	90.0 ± 2.8	72.3 ± 3.0*	73.4 ± 1.0*	66.1 ± 3.2*
Caloric intake (kcal)	349.5 ± 16.4	313.1 ± 5.5	342.2 ± 10.7	391.4 ± 15.8	381.9 ± 5.4	343.5 ± 5.3
Body mass gain (g)	230.3 ± 10.1	225.6 ± 6.2	239.3 ± 10.1	317.1 ± 8.8*	282.7 ± 11.8*	268.4 ± 9.9^#^
Liver (g/100 rat mass)	2.55 ± 0.09	2.31 ± 0.10	2.66 ± 0.07	2.47 ± 0.07	2.34 ± 0.04	2.45 ± 0.06
Adiposity index	7.05 ± 0.3	4.98 ± 0.4	6.54 ± 0.5	10.96 ± 0.1*	7.89 ± 0.57^#^	8.75 ± 0.35^#^
n	6–10	6–10	6–10	6–10	6–10	6–10

*p < 0.05 compared to CT/E group. ^#^p < 0.05 compared to HF/E group. (ANOVA two way followed by Tukey’s test), expressed as mean values ± standard error of the mean. n = number of animals. Caloric intake = food intake × kcal provided by each diet. Gain mass body = final body mass − initial body mass. Adiposity index [absolute mass (g) deposits of inguinal fat + retroperitoneal + epidydimal/rat mass (g) × 100]. n = number of animals.

### Oral A-1317 Treatment Improves Total Cholesterol Plasma Levels and Liver Damage

HF/E rats showed increased plasma levels of leptin, total cholesterol, triglycerides and ALT compared to CT/E rats. Treatment with Ang-(1–7) or A-1317 decreased leptin and triglyceride levels compared to HF/E rats. In addition, only treatment with A-1317 decreased total cholesterol, ALT, and AST levels compared to HF/E rats (**Table 5**).

**Table 5:** Biochemical parameters of rats fed with CT or HF for 15 weeks and treated with HP*β*CD/A-1317 or HP*β*CD/Ang-(1–7) during the last 7 weeks of the diet.

Parameters	Experimental Groups					
	CT/E	CT/Ang-(1–7)	CT/A-1317	HF/E	HF/Ang-(1–7)	HF/A-1317
Leptin (ng/dl)	5.25 ± 1.35	5.55 ± 1.14	7.65 ± 1.42	19.40 ± 1.40*	6.66 ± 0.63^#^	4.92 ± 0.27^#^
Total cholesterol (mg/dl)	40.2 ± 3.2	51.8 ± 3.5	47.2 ± 1.9	74.5 ± 7.2*	55.9 ± 5.2	50.5 ± 5.6^#^
Triacylglycerol (mg/dl)	37.2 ± 4.9	48.1 ± 4.9	49.1 ± 5.0	68.2 ± 5.9*	40.7 ± 4.5^#^	43.4 ± 7.9^#^
ALT (U/L)	66.6 ± 2.0	75.6 ± 15.0	64.1 ± 4.5	126.3 ± 28.1*	72.6 ± 3.6	62.7 ± 1.6^#^
AST (U/L)	174.9 ± 13.5	133.4 ± 19.8	140.2 ± 12.6	219.4 ± 17.6	171.3 ± 17.6	151.8 ± 6.4^#^
Creatinine (mg/dl)	0.575 ± 0.04	0.637 ± 0.03	0.600 ± 0.02	0.675 ± 0.02	0.575 ± 0.02	0.588 ± 0.01
Albumin (g/dl)	3.34 ± 0.08	3.47 ± 0.06	3.30 ± 0.10	3.46 ± 0.06	3.47 ± 0.08	3.32 ± 0.10
Total proteins (g/dl)	8.06 ± 0.12	7.71 ± 0.49	6.65 ± 0.90	7.32 ± 0.10	7.21 ± 0.13	7.49 ± 0.18
n	8	8	8	8	8	8

*p < 0.05 compared to CT/E group. **^#^**p < 0.05 compared to HF/E group. (ANOVA two way followed by Tukey’s test), expressed as mean values ± standard error of the mean. n = number of animals.

### Oral A-1317 Treatment Improves Insulin Resistance by Increasing *β*-Cells’ Functional Capacity

**Figure 1:**
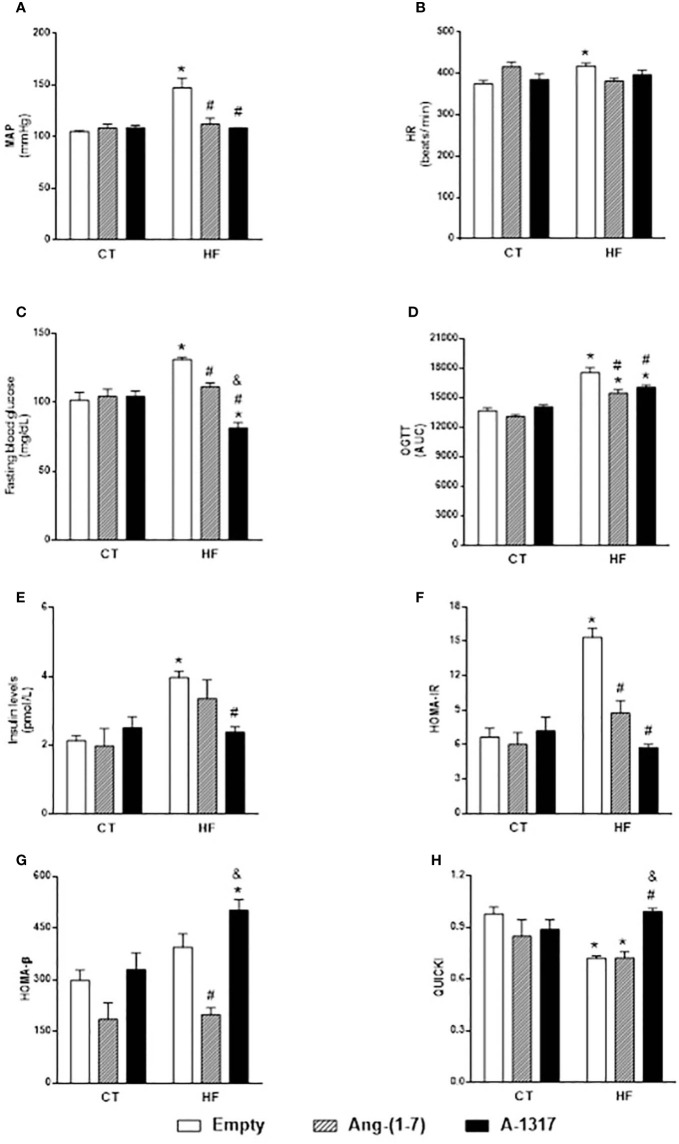
Evaluation of hemodynamic parameters and insulin resistance. Evaluation of mean blood pressure (MAP, mmHg, **A**), heart rate (HR, beats/min, **B**), Fasting blood glucose (mg/dL, **C**), area under the curve (AUC) of blood glucose during oral glucose tolerance test (OGTT; **D**), Insulin levels (pmol/L; **E**), model assessment of IR homeostasis (HOMA-IR, **F**) = [fasting insulin (FI) x fasting glucose (FG) / 22.5], homeostasis evaluation model of the functional capacity of β-cells (HOMA-β, **G**) = (20 x FI) / (FG 3.5), quantitative insulin sensitivity check index (QUICKI, **H**) = 1/[LOG(FI)/LOG(FG)] in rats fed with high-fat (HF) or control (CT, n=6-8) diet for 15 weeks and treated with empty (HP*β*CD), Ang-(1-7) or A-1317 during the last 7 weeks of diets. Values are expressed as mean ± SEM and analyzed using two-way analysis of variance (ANOVA) followed by Tukey´s post-test.*p<0.05 compared to CT/E group, ^#^p<0.05 compared to HF/E group, ^&^p<0.05 compared to HF/Ang-(1-7) group.

HF/E rats presented increased MAP and HR, fasting blood glucose, plasma insulin levels, OGTT, HOMA-IR and decreased QUICKI compared to CT/E rats (**Figures 1A–H**). Treatment with Ang-(1–7) and A-1317 decreased MAP, HOMA-IR compared to CT/E rats and OGTT compared to CT/E and HF/E rats (**Figures 1A, D, F****)**. In addition, HF/Ang-(1–7) rats showed decreased fasting blood glucose and HOMA-*β* compared to HF/E rats and QUICKI compared to CT/E rats (**Figures 1C, G, H****)**. Only, treatment with A-1317 decreased insulin levels compared to HF/E rats and fasting blood glucose compared to CT/E, HF/E, HF/Ang-(1–7) and increased HOMA-*β* compared to CT/E and HF/Ang-(1–7) rats and QUICKI compared to HF/E and HF/Ang-(1–7) rats (**Figures 1C, E, G, H****)**.

### Oral A-1317 Treatment Increases *Mas* and *MRGD* mRNA Gene Expression in the Liver

**Figure 2:**
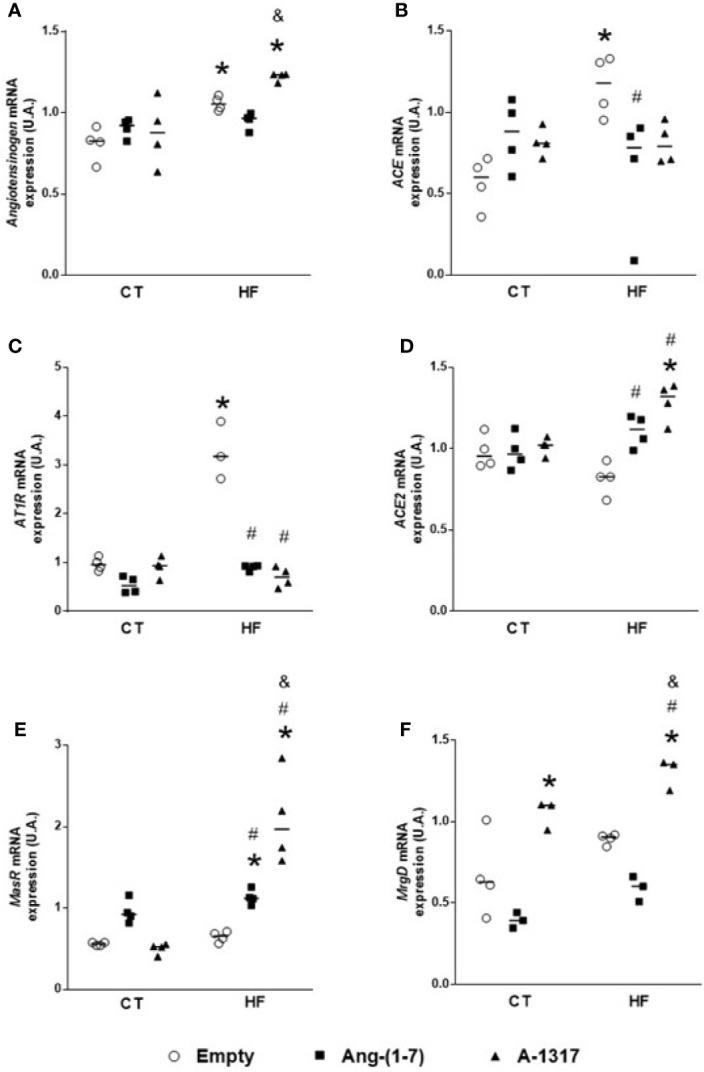
Evaluation of the mRNA components expressions of the renin angiotensin system in the live. *Angiotensinogen* **(A)**, *angiotensin-converting enzyme* (*ACE*, **B**), *angiotensin II receptor type 1* (*AT1R*, **C**), *angiotensin-converting enzyme 2* (*ACE2*, **D**), *Mas-related G protein–coupled receptor* (*Mas*, **E**), *Mas-related G protein-coupled receptor member D* (*MRGD*, **F**) mRNA gene expressions the in liver of rats fed with high-fat (HF, n=4) or control (CT, n=4) diet for 15 weeks and treated with empty (HP*β*CD), Ang-(1-7) or A-1317 during the last 7 weeks of diets. Values are expressed as mean ± SEM and analyzed using two-way analysis of variance (ANOVA) followed by Tukey´s post-test. *p<0.05 compared to CT/E group, ^#^p<0.05 compared to HF/E group, ^&^p<0.05 compared to HF/Ang-(1-7) group.

HF/E rats increased *angiotensinogen, ACE*, *AT1R* mRNA gene expression compared to CT/E rats (**Figures 2A–C**). However, treatment with Ang-(1–7) or A-1317 decreased A*T1R* mRNA gene expression compared to CT/E rats. In addition, treatment with Ang-(1–7) decreased *ACE* mRNA gene expression and increased *ACE2* mRNA gene expression compared to HF/E and *Mas* mRNA gene expression compared to CT/E and HF/E (**Figures 2B–E**). Furthermore, the treatment with A-1317 increased *angiotensinogen* mRNA gene expression compared to CT/E and HF/Ang-(1–7) and *ACE2* mRNA gene expression compared to CT/E and HF/E. Finally, treatment with A-1317 increased *Mas* and *MRGD* mRNA gene expression compared to CT/E, HF/E, and HF/Ang-(1–7) (**Figures 2A, D–F**).

### Oral A-1317 Treatment Decreases Redox Process in the Liver

**Figure 3:**
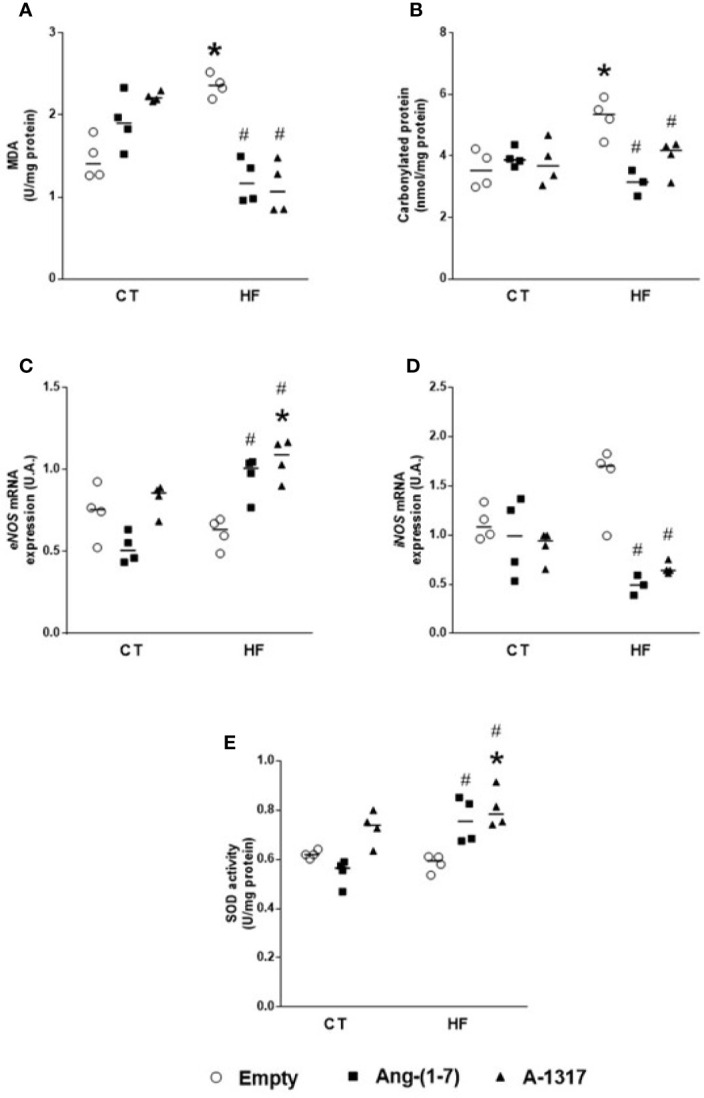
Evaluation of oxidative stress in the live. Concentrations of malondialdehyde (MDA, U/mg of protein) by concentrations of thiobarbituric acid-reactive substances (TBARS) method **(A)** carbonylated protein **(B)**, *endothelialnitric oxide synthase (eNOS)* mRNA gene expression **(C)**, *indisible nitric oxide synthase (iNOS)* mRNA gene expression **(D)**, superoxide dismutase (SOD, **E**) in the liver of rats fed with high-fat (HF, n=4 ) or control (CT, n=4) diet for 15 weeks and treated with empty (HP*β*CD), Ang-(1-7) or A-1317 during the last 7 weeks of diets. Values are expressed as mean ± SEM and analyzed using two-way analysis of variance (ANOVA) followed by Tukey´s post-test. ^*^p<0.05 compared to CT/E group, ^#^p<0.05 compared to HF/E group, & p<0.05 compared to HF/Ang-(1-7) group.

HF/E rats showed increased concentrations of MDA and carbonyl protein compared to CT/E rats (**Figures 3A, B****)**. Treatment with Ang-(1–7) or A-1317 decreased concentrations of MDA, carbonyl protein, and *iNOS* mRNA gene expression compared to HF/E rats and increased *eNOS* mRNA gene expression and SOD activity compared to HF/E. However, treatment with A-1317 increased *eNOS* mRNA gene expression and SOD activity compared to CT/E rats, too (**Figures 3C–E**).

### Oral A-1317 Treatment Increases *AMPK* mRNA Gene Expression in the Liver

**Figure 4:**
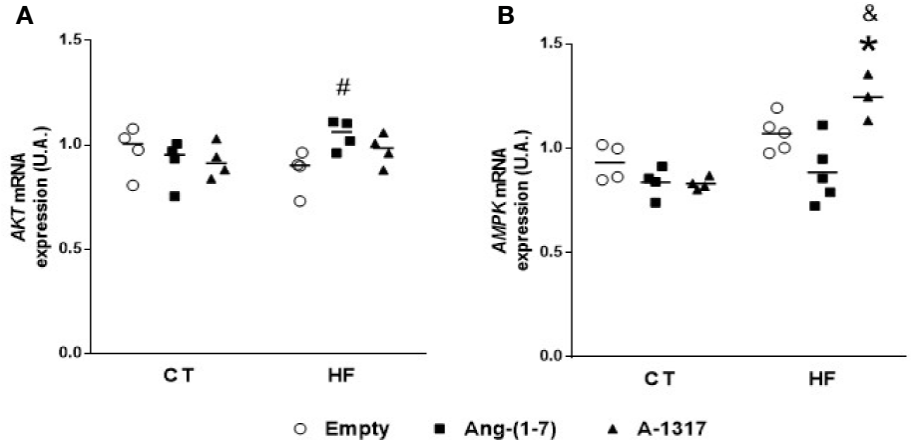
mRNA expressions of protein kinases in the liver. *Protein kinase B* (*AKT*, **A**), *adenosine monophosphate activated protein kinase* (*AMPK*, **B**) mRNA gene expressions in the liver of rats fed with high-fat (HF, n=3-4) or control (CT, n=4) diet for 15 weeks and treated with empty (HP*β*CD), HPβCD/Ang-(1-7) or HPβCD/A-1317 during the last 7 weeks of diets. Values are expressed as mean ± SEM and analyzed using two-way analysis of variance (ANOVA) followed by Tukey´s post-test. *p<0.05 compared to CT/E group, ^#^p<0.05 compared to HF/E group, ^&^p<0.05 compared to HF/Ang-(1-7) group.

Treatment with Ang-(1–7) increased *AKT* mRNA gene expression compared to HF/E rats (**Figure 4A**). On the other hand, treatment with A-1317 did not change *AKT* mRNA gene expression but increased *AMPK* mRNA gene expression compared to CT/E and HF/Ang-(1–7) rats (**Figure 4B**).

## Discussion

In the present study, Ang-(1–7) and A-1317 oral treatments in MetS rats have similar beneficial effects on glucose metabolism and blood pressure. However, oral treatment with the A-1317 compound was more efficient in restoring IR by ameliorating pancreatic *β* cell functionality. In addition, Ang-(1–7) increased *Mas* and *AKT* liver mRNA gene expression while A-1317 increased both, *Mas* and *MRGD*, and *AMPK*. Taken together, our data showed that treatment with A-1317 was able to ameliorate MetS disorder probably by Mas and MRGD *via* activation of AMPK and increasing pancreatic *β* cell function.

Both Ang-(1–7) and A-1317 oral treatment in MetS rats, besides maintaining similar caloric intake, improved metabolism by reducing adiposity index, fasting blood glucose, OGTT, HOMA-IR, triglycerides, lipid peroxidation, AT1R, iNOS liver mRNA gene expression and carbonylated protein and MAP and increased eNOS and ECA2 liver mRNA gene expression and SOD activity. These beneficial effects induced by oral treatment with Ang-(1–7) in MetS rats are in agreement with previous studies from our laboratory using the same experimental protocol (Barbosa et al., 2019; Figueiredo et al., 2019) and in the literature studies in prevention of MetS (Giani et al., 2009; Oliveira Andrade et al., 2014; Santos et al., 2014; Liu et al., 2019). However, A-1317 peptide was more effective than Ang-(1–7) in reducing body mass gain, ALT, AST, total cholesterol, insulin, fasting blood glucose and in increasing *AMPK* mRNA gene expression, and *β* cell capacity showed by enhancing HOMA-*β* and QUICKI (Sarafidis et al., 2007), whereas Ang-(1–7) does not change insulin levels and *AMPK* mRNA gene expression, reduced HOMA-*β* and QUICKI and increased *AKT* mRNA gene expression in the liver. This reduction in plasma insulin levels induced by A-1317 treatment may be due to the action of AMPK on direct translocation of glucose transporter type 4 (GLUT-4) to membrane and/or by regulating, positively, insulin receptor substrate 1 (Harrington et al., 2004; Shah et al., 2004) and/or by inhibiting insulin *via* (mTOR) (Fisher, 2006; Inoki et al., 2012) and therefore improving insulin sensitivity and making glucose levels lower. In fact, in the present study, treatment with A-1317 was more effective in reducing plasma glucose levels compared to treatment with Ang-(1–7). In addition, both insulin and activated AMPK suppress the expression of gluconeogenic enzymes (Lochhead et al., 2000). The molecular mechanisms that lead to the onset of IR are not completely understood. However, it is clear that there is a cross-talk between insulin and Ang II and hormones such as leptin (Coppari and Bjørbaek, 2012), which in the present study was shown by reduction of plasma leptin levels and *AT1R* hepatic mRNA gene expression induced by both Ang-(1–7) and A-1317 treatments. Additionally, activated AMPK stimulates the catabolic pathways (Hardie et al., 2012), phosphorylates and inhibits acetyl coenzyme A carboxylase and 3-hydroxy 3-methylglutaryl coenzyme A reductase, which are the limiting enzymes of fatty acid and cholesterol biosynthesis, respectively (Lin and Hardie, 2018). Furthermore, AMPK promotes glucose metabolism homeostasis by decreasing free fatty acid concentration and consequently reducing ectopic fat accumulation, preventing hepatic steatosis and the formation of reactive species (Qiu et al., 2014). According to the present study treatment with A-1317, and not treatment with Ang-(1–7), reduced cholesterol plasma levels. Literature data also show that AMPK activity correlates with increased antioxidant enzyme activity (Qiu et al., 2014; Hinchy et al., 2018). Thus, A-1317, and not Ang-(1–7), probably because it acts *via* AMPK, becomes more efficient in restoring IR, liver damage and improving both glucose and cholesterol metabolism.

Studies show that circulating Ang-(1–7) improves glucose and lipid metabolism by ameliorating insulin pathway and redox process in the liver and gastrocnemic muscle in rats fed with diet H (Oliveira Andrade et al., 2014; Williams et al., 2016; Figueiredo et al., 2019) and fed with fructose diet (Giani et al., 2009; Muñoz et al., 2012; Marcus et al., 2013). In the present study, oral treatment with both Ang-(1–7) and A-1317 improved the redox process in the liver by increasing *eNOS* mRNA gene expression and SOD activity and reducing MDA, carbonylated protein and *iNOS* mRNA gene expression compared to HF/E rats. However, oral treatment with A-1317 increased *eNOS* mRNA gene expression and SOD activity also compared to CT/E rats and thus, the increase in the *AMPK* mRNA gene expression may have favored the *eNOS* mRNA gene expression and SOD activity (García-Prieto et al., 2019), which together, may have contributed to the restoration of liver damage observed by normalization of ALT and AST levels that was shown only in the treatment with A-1317.

Although most studies show that Ang-(1–7) acts through AKT-dependent Mas and NO (Sampaio et al., 2007; Muñoz et al., 2012), some studies suggest that Ang-(1–7) may also act through MRGD that shows high Mas homology (Tetzner et al., 2016; Gunarathne et al., 2019). Our data from the present study showed that Ang-(1–7) at MetS does not act through the MRGD but through the Mas, stimulating eNOS activation and probably NO production and reducing protein and lipid oxidation and increasing SOD activity dependent to AKT (Patel et al., 2016). Furthermore, studies suggest that *ECA2* mRNA gene expression may be regulated by energy stress and AMPK activation (Patel et al., 2016), besides decreasing the mRNA gene expression of *ACE* (Kohlstedt et al., 2011; Oliveira Andrade et al., 2014; Liu et al., 2019). In the present study, both oral treatment with Ang-(1–7) and A-1317 increased liver *ECA2*, *Mas* and reduced *AT1R* mRNA gene expression compared with HF/E rats. However, oral treatment with A-1317 also increased *angiotensinogen*, *Mas*, *MRGD*, and *AMPK* mRNA gene expression in both CT/E and HF/Ang-(1–7) rats and did not increase *AKT* mRNA gene expression in the liver. These data suggest that although both Ang-(1–7) and A-1317 act on the ECA2/Ang-(1–7)/Mas axis, A-1317 has affinity for both *Mas* and *MRGD*, but this new compound, acts preferentially *via* AMPK, while Ang-(1–7) appears to act primarily on Mas *via* AKT in MetS rats. Further studies are needed to confirm these findings.

## Conclusion

Our data together show that oral treatment with A-1317 induces similar benefits to treatment with Ang-(1–7) on metabolic disturbances and on blood pressure in rats with MetS. However, oral treatment with A-1317 was more efficient in reducing body mass gain, improving IR, liver damage and *β*-cell functionality.

## Data Availability Statement

The raw data supporting the conclusions of this article will be made available by the authors, without undue reservation, to any qualified researcher.

## Ethics Statement

The project was approved by the animal ethics committee of the Federal University of Ouro Preto protocol 2016/51.

## Author Contributions

ACA and RASS conceived and designed the experiment, analyzed the data, and wrote the manuscript. CMB, TCL, MAB, and ACA performed the experiments, analyzed the data, and wrote the manuscript. All authors contributed to the article and approved the submitted version.

## Conflict of Interest

The authors declare that the research was conducted in the absence of any commercial or financial relationships that could be construed as a potential conflict of interest.

## Abbreviations

MetS, metabolic syndrome; NO, nitric oxide; HF, high fat; CT, control; HP*β*CD, hydroxypropyl-*β*-cyclodextrin; E, empty; RAS, renin angiotensin system; ALT, alanine aminotransferase; AST, aspartate aminotransferase; OGTT, oral glucose tolerance test; IR, insulin resistance; HOMA-IR, model assessment of IR homeostasis; HOMA-*β* model for assessing homeostasis of the functional capacity of the *β* cells; MAP, mean arterial pressure; HR, heart rate; AT1R, angiotensin II type 1 receptor; ACE2, angiotensin-converting enzyme 2; Ang II, angiotensin II; Ang-(1–7), angiotensin-(1–7); Mas, Mas-related G protein–coupled receptor; MRGD Mas-related G protein-coupled receptor member D; MDA, malondialdehyde; iNOS, indisible nitric oxide synthase; SOD, superoxide dismutase; QUICKI, quantitative insulin sensitivity check index; AKT, protein kinase B; AMPK, adenosine monophosphate activated protein kinase; GLUT-4, glucose transporter type 4; AUC, area under the curve; LDL, low-density lipoprotein, HDL, high-density lipoprotein; NADPH oxidase, nicotinamide adenine dinucleotide phosphate oxidase; PI3K, phoshoinositide 3-kinase; PFK-1, Phosphofructokinase-1; KATP, adenosine triphosphate sensitive potassium channels; TBARS, thiobarbituric acid reactive substance; LAPAC, Pilot Laboratory of Clinical Analyses.
